# A case–control study of the impact of the East Anglian breast screening programme on breast cancer mortality

**DOI:** 10.1038/sj.bjc.6604123

**Published:** 2007-12-04

**Authors:** P C Allgood, J Warwick, R M L Warren, N E Day, S W Duffy

**Affiliations:** 1 Cancer Research UK Department of Epidemiology, Mathematics and Statistics, Wolfson Institute of Preventive Medicine, Charterhouse Square, London EC1M 6BQ, UK; 2 Department of Radiology, Addenbrooke's Hospital, Cambridge CB2 2QQ, UK; 3 Strangeways Research Laboratory, Wort's Causeway, Cambridge CB1 8RN, UK

**Keywords:** breast cancer mortality, screening, case–control

## Abstract

Although breast cancer screening has been shown to work in randomised trials, there is a need to evaluate service screening programmes to ensure that they are delivering the benefit indicated by the trials. We carried out a case–control study to investigate the effect of mammography service screening, in the NHS breast screening programme, on breast cancer mortality in the East Anglian region of the UK. Cases were deaths from breast cancer in women diagnosed between the ages of 50 and 70 years, following the instigation of the East Anglia Breast Screening Programme in 1989. The controls were women (two per case) who had not died of breast cancer, from the same area, matched by date of birth to the cases. Each control was known to be alive at the time of death of her matched case. All women were known to the breast screening programme and were invited, at least once, to be screened. There were 284 cases and 568 controls. The odds ratio (OR) for risk of death from breast cancer in women who attended at least one routine screen compared to those who did not attend was 0.35 (CI: 0.24, 0.50). Adjusting for self-selection bias gave an estimate of the breast cancer mortality reduction associated with invitation to screening of 35% (OR=0.65, 95% CI: 0.48, 0.88). The effect of actually being screened was a 48% breast cancer mortality reduction (OR=0.52, 95% CI: 0.32, 0.84). The results suggest that the National Breast Screening Programme in East Anglia is achieving a reduction in breast cancer deaths, which is at least consistent with the results from the randomised controlled trials of mammographic screening.

Randomised trials have shown that breast screening reduces breast cancer mortality (Jonsson *et al*, 2000, 2001, 2003; Duffy *et al*, 2002; Nyström *et al*, 2002; Tabar *et al*, 2003). Whether this is achieved in routine practice requires monitoring as certain factors might alter the effect. These include technical changes in the mammographic equipment since the trials, different screening frequencies to those of the trials, expertise of the professionals involved, and quality of the assessment process. For this reason, quality assurance is a fundamental part of the National Health Service Breast Screening Programme (NHSBSP), which aims at minimum standards and improved performance so that women have access to high-quality screening service wherever they live (NHS, 2006).

The case–control design is convenient and its basic principle here is simple: women who have died from the disease are selected as the cases and for each, we select as controls a number of age-matched women (usually one or two) known to be alive at the time of death of the case. We then compare the screening histories of cases and controls prior to the diagnosis dates of the cases. If the screening is reducing mortality from the disease, the cases will tend to have lesser screening histories than the controls.

The design is prone to certain biases, in particular, self-selection bias, since women who accept the invitation to screening may have better health status *a priori* than those who do not, conferring a reduced likelihood of dying from the disease independent of screening. This is a bias in favour of screening, and a method for correcting it is given by Duffy *et al* (2002).

We therefore conducted a case–control study to

estimate the effect of the NHSBSP on breast cancer mortality in the East Anglian region of the UK, adjusting for self-selection bias; and
ascertain whether the reduction in breast cancer mortality being achieved is consistent with that observed in the randomised screening trials.

## MATERIALS AND METHODS

All female residents of the East Anglian region (as delineated when screening began) aged 50–70 years and registered with a general practitioner were invited to attend breast screening once in every 3 years. The NHSBSP was established in the counties of Norfolk, Suffolk, and Cambridgeshire in 1989.

For sample size, assuming a relative risk of 0.68 (Cuzick *et al*, 1997), and a 70% coverage, 264 cases and 528 matched controls would detect a significant effect with 80% power at the 5% level with a two-sided test (Breslow and Day, 1987). We therefore aimed for 300 cases and 600 controls as a fail-safe measure. Cases were deaths from female breast cancer in women aged 50–70 years at diagnosis from 1995 onwards, to avoid the accumulation found in the prevalent round of the screening programme, and to minimise screening opportunity bias (Walter, 2003). Deaths were included for which breast cancer was given as the primary or a contributory cause on the death certificate.

We excluded those women who had not been invited to breast screening at least once. Cases were taken at random from the EA cancer registry database. There were two controls for each case and matched by date of birth, most coming from the same health authority. The controls were all alive at the time of death of their case and were also known to the data source before the diagnosis of the case. Controls were obtained from the National Health Service (NHS) Exeter System database and individual screening units. Each control was allocated a pseudodiagnosis date equal to the date of diagnosis of that control's matched case. The following information was obtained:

- date of birth,
- date of diagnosis (if a case),
- stage at diagnosis (if a case),
- date of death (if a case), and
- complete screening history including dates of invitations and attendances, and outcomes of attendances.

### Statistical analysis

The data were analysed by conditional logistic regression. The primary outcome of whether a woman had ever attended breast screening prior to diagnosis/pseudodiagnosis is potentially subject to self-selection or healthy volunteer bias, as it compares women who choose to take up the invitation to screening with women who do not. The latter may have poorer health *a priori* and therefore be more likely to die of the disease, independent of screening. An adjustment can be made for this using the relative rate of breast cancer deaths in non-attenders in the randomised trials compared to the trial controls (Duffy *et al*, 2002).

As an alternative, we adjusted for socio-economic status as measured by the index of multiple deprivation (IMD) derived from the womens’ postcodes, in the logistic regression, as this is the main confounding factor of outcome and attendance. The IMD is an area-based measure of relative deprivation, based on census statistics for overcrowded housing and other factors. Secondary analyses included the time since last screen attended, age at first attendance, and the number of screens attended.

## RESULTS

Out of the 300 cases identified, 16 were excluded because they were diagnosed while awaiting their first screening invitation. The remaining 284 were matched on birth date to two controls each, giving a total study population of 852 women.

Slightly more controls than cases were in the two most affluent deprivation groups 1 and 2 (51 *versus* 48%) and slightly fewer in groups 3 and 4, but this was not significant (*P*=0.3). The average age at diagnosis/pseudodiagnosis was 60.7 years (range 50.4–70.5) for both cases and controls as expected due to the matching. Mean age at first screen was 55.3 for cases and 55.5 for controls (Table 1). The mean number of screens was 1.39 for the cases compared with 1.70 for the controls, a significant difference (*P*<0.001).

More than 85% (242/284) of the cases presented symptomatically, 58% as interval cancers and 27% in women who had never attended screening. As might be expected with fatal cancers, the tumours were at relatively advanced stages, with 49% being at TMN stage 2 and 37% at stage 3 or 4. The average age at death was 63.0 years (range 50.8–77.6) and average time from diagnosis to death was 2.4 years, ranging from 3 days to 8.8 years. The years of diagnosis and death ranged from 1995 to 2004.

The unadjusted OR of women who attended at least one routine screen compared to those who never attended screening was 0.35 (95% CI: 0.24, 0.51; *P*<0.001) (Table 2). Adjusting for self-selection bias using the more conservative intention-to-treat analysis (Duffy *et al*, 2002) gave a 35% estimated mortality reduction (OR=0.65, 95% CI: 0.48, 0.88; *P*=0.005). Actually being screened, adjusted for self-selection bias, was associated with a 48% reduction (OR=0.52, 95% CI 0.32, 0.84; *P*=0.007). As an alternative to self-selection bias correction, adjustment for IMD had no effect on the OR associated with ever being screened (OR=0.35, 95% CI 0.23–0.51, *P*<0.001).

The major effect of any screens attended is an approximately 60% lower risk of death from breast cancer compared to those who had attended none; there was no significant effect for number of screens attended. Risk was higher in those with a screen within 1 year than those whose last screen was between 1 and 2 years prior to diagnosis or pseudodiagnosis, due to deaths from screen-detected cancers. Thereafter, risk increased with increasing time since last screen. Risk was higher in those whose first screen took place at age 52 or more compared to age 51 or less, but the trend was not significant, possibly due to the small numbers.

## DISCUSSION

The results of this study are encouraging. After adjusting for self-selection bias, we found a significant 35% reduction in breast cancer mortality in association with invitation to screening and a significant 48% reduction with actually being screened, if anything, exceeding the benefits in the randomised trials (Smith *et al*, 2004). However, the correction method makes an important assumption that the relative mortality for non-compliers compared with a population not invited for screening is the same in the programme in question as in the randomised trials. From the available information (the Swedish Two-county, Malmö, Gothenburg, Stockholm and Canadian trials) (Duffy *et al*, 2002), non-attenders had a 36% higher risk of breast cancer mortality than uninvited controls. This may not hold for our population, but it seems likely that there is at least some self-selection bias, and the uncorrected estimates of the mortality reduction seem implausible.

There is a suggestive increase in risk with first screen at 52 or over, suggesting that there is an advantage to a first screen at the lower age limit of 50, though the trend was not significant; it should be examined in other studies.

Adjustment for IMD made no difference to the estimated effect of screening on risk, perhaps because although socioeconomic status is strongly associated with screening in urban areas, it may not be so in rural areas. In East Anglia, IMD was not strongly related to screening exposure, around 84% regardless of IMD.

There was no substantial trend in risk with number of screens attended, consistent with the fact that only the screen at which a cancer is detected actually confers a benefit (Elmore *et al*, 2005). Aside from a high risk in those very recently screened, there was an increase in the risk with time since last screen. The high risk in those screened in the last year could be due to screen-detected cancers. When these are excluded, the ORs for 1–2, 2–4, and 4 or more years since last screen, relative to less than 1 year, were 1.05, 1.32, and 1.49 respectively.

From other case–control screening studies, there is a range of results from a very small benefit to large reductions in mortality as observed in this study (Palli *et al*, 1989; Mittenburg *et al*, 1998; Fielder *et al*, 2004; Elmore *et al*, 2005; Gabe *et al*, 2007). Our estimated benefit may be slightly biased in favour of screening by the selection of recently diagnosed cases (1995–2004), which will of necessity be relatively rapidly fatal and therefore may under-represent screening-exposed fatal cancers. Restricting analysis to those diagnosed before 1999, we obtain an uncorrected mortality reduction of 59% with ever being screened, and self-selection bias corrected estimates of 27% for invitation to screening, and 37% for actually being screened.

In conclusion, this study shows strong evidence that the NHS breast screening programme in East Anglia is achieving at least a 30% reduction in breast cancer mortality in women attending screening, consistent with the randomised trials. We recommend expanding the case–control study to other regions of the UK and internationally.

## Figures and Tables

**Table 1:** Patient characteristics, screening history by case–control status

	**Cases**	**Controls**
Total numbers	284	568
		
*Index of multiple deprivation (IMD), N (%)* a		
1st quartile	75 (26)	135 (25)
2nd quartile	61 (22)	146 (26)
3rd quartile	68 (24)	139 (25)
4th quartile	80 (28)	132 (24)
		
Mean age at diagnosis or pseudodiagnosis (range)	60.7 (50.4–70.5)	60.7 (50.4–70.5)
		
*Age group, N (%)*		
50–54	50 (18)	100 (18)
55–59	85 (29)	170 (29)
60–64	67 (24)	134 (24)
65+	82 (29)	164 (29)
		
Mean age at first screen, years (range)	55.3 (45.2–65.4)	55.5 (43.5–65.5)
		
*Number of screening visits, N (%)*		
0	77 (27)	63 (11)
1	87 (31)	185 (32)
2	66 (23)	198 (35)
3	45 (16)	106 (19)
4+	10 (3)	16 (3)
		
Mean number of screens (range)	1.39 (0–5)	1.70 (0–5)
		
*Outcome of first screen, N (%)*		
Routine recall	190 (92)	498 (99)
Recall to assessment and benign	4 (2)	7 (1)
Recall to assessment and cancer	13 (6)	0 (0)
		
*Mode of detection, N (%)*		
Non-attender	77 (27)	
First screen (screen detected)	10 (3)	
Subsequent screen (screen detected)	32 (12)	
Interval cancer	165 (58)	
		
*TMN stage, N (%)* b		
1	37 (14)	
2	137 (49)	
3	25 (9)	
4	76 (28)	
		
Mean age at death, years (range)	63.0 (50.8–77.6)	

a 16 (2.8%) controls had missing IMD.

b 9 (3.2%) cases had missing TMN stage.

**Table 2:** Odds ratios for risk of death from breast cancer by screening history 1995–2004

**Screening factors**	**No. of cases/controls**	**Odds ratio (95% CI)**
*Ever attended*		
Never	77/63	1
Ever	207/505	0.35 (0.24, 0.50)
Self-selection corrected (invitation effect)		0.65 (0.48, 0.88)
Self-selection corrected (screening effect)		0.52 (0.32, 0.84)
		
*Number of screens attended*		
1	86/185	1
2	66/198	0.70 (0.43, 1.11)
3+	55/122	1.03 (0.59, 1.77)
None	77/63	2.51 (1.56, 4.03)
		
*Time since last screen*		
<1 year	65/113	1
1<2 years	41/121	0.43 (0.24, 0.77)
2<4 years	62/175	0.48 (0.28, 0.81)
4+ years	39/96	0.55 (0.29, 1.04)
Never screened	77/63	1.71 (1.03, 2.80)
		
*Age at first screen (screened women only)*		
<52	53/144	1
52–53	50/91	2.38 (1.02, 5.57)
54<58	60/137	2.25 (0.83, 6.09)
>58	44/133	1.54 (0.33, 7.01)
